# Strobe training as a visual training method that improves performance in climbing

**DOI:** 10.3389/fspor.2024.1366448

**Published:** 2024-05-20

**Authors:** Antonia Ioana Vasile, Monica Iulia Stănescu

**Affiliations:** Doctoral School, National University of Physical Education and Sports, Bucharest, Romania

**Keywords:** strobe training, visual training, climbing performance, cognition in climbing, cognitive agility, spatial orientation, sensory reweighting, strobe glasses

## Abstract

**Introduction:**

Strobe training is a form of visual training where the athlete has to practice during intermittently dark conditions. Strobe training improves visual, perceptual, and cognitive skills, which will enhance athletic performance. Strobe training can influence multiple training components in climbing: psychological, tactical, physical, and technical training.

**Materials and methods:**

The study was conducted on 17 elite climbers from Romania (10 male and 7 female), representing the entire National Youth Climbing Team. The research group was divided into a control group (*n* = 8) and an experimental group (*n* = 9). The used instruments were the Cognitrom battery (for cognitive skills, such as spatial skills and reactivity), the Witty SEM system (for motor-cognitive skills, such as cognitive agility, visual processing speed, and visual memory), and the International Rock Climbing Research Association (IRCRA) performance-related test battery for climbers (climbing-specific motor skills). The experimental group had 20 strobe training sessions, which took place during one calendar year, as an additional session to their climbing schedule done with their principal trainer. The strobe session was once a week, depending on the periodization of the macrocycle (preparatory, competitional, and transition periods). The control group and the experimental group had similar climbing training sessions during the 1-year macrocycle in terms of intensity and volume of their training.

**Results:**

Strobe training improved on-sight performance (*d* = 0.38) and red-point performance (*d* = 0.36). Strobe training improved the majority of cognitive skills [all spatial skills (*d* = 1.27 for mental image transformation; *d* = 1.14 for spatial orientation; *d* = 1.59 for image generation) and simple reaction time (*d* = 0.99)]. Strobe training improved all motor-cognitive skills (*d* = 0.16 for visual memory; *d* = 1.96 for visual memory errors; *d* = 1.39 for visual processing speed; *d* = 1.94 for visual processing errors; *d* = 1.30 for cognitive agility). Strobe training improved many climbing-specific parameters (flexibility and upper body strength) (*d* = 0.44 and *d* = 0.47 for flexibility parameters; *d* = 0.50 to 0.73 for upper body strength parameters).

**Discussion:**

Strobe training is an effective training method for enhancing performance that should be used on more experienced climbers. It acts more on spatial skills, rather than on reactivity skills, developing the visual-motor coordination system. Strobe training has greater effects on climbers aged below 16 years, as youth athletes rely more on visual input compared to adults. The improvement in climbing-specific variables was due to the additional climbing session done weekly. Strobe training acts more on the cognitive component of training than on the motor component of training in climbing.

## 1 Introduction

Sport climbing was included in 2020 at the Olympic Games and has grown significantly over the last years, both as a recreational activity as well as a competitive activity (1). Sport climbing has three disciplines: lead climbing (with rope protection on longer routes), bouldering (with mattress floor protection on shorter routes), and speed climbing (on a standardized route with standardized holds) (2). Climbing performance can be considered as follows: on-sight (from the first try, without additional information about the route), flash (from the first try, with additional information), or red-point (after multiple tries) (3).

The visual analyzer is responsible for analyzing approximately 80% of the sensory stimuli that come to the brain (4). Multiple pathways in the central nervous system process visual stimuli to determine what the eyes see so that the brain can analyze the objects in the surrounding space. This visual-spatial analysis is a critical skill needed in numerous sports (4). The body's analyzers are cutaneous, kinesthetic, visual, acoustic, vestibular, olfactory, and gustatory (5). For postural control, the central nervous system counts on the feedback from cutaneous, kinesthetic, visual, and vestibular analyzers (5). The sensations analyzed by the four analyzers provide an internal model for recognizing body position and movement in relation to the external environment (6). Visual training implies training the ability to shift the visual field to increase performance, by activating different brain areas (7). Moreover, it was suggested that the control of lower limbs takes place in the posterior regions of the brain dorsal stream, in the right hemisphere, whereas the control of upper limbs takes place in the anterior regions of the brain, involving both hemispheres (7). Elite climbers have better visual perception compared to advanced climbers in terms of visual field, but not in terms of visual acuity and contrast sensitivity (8). This result is explained by climbing experience from two points of view: first, because of the greater time spent training the visual system; and second, because of the greater complexity of the stimuli that elite climbers get from climbing harder routes where holds are less perceptible (8).

Sensory weighting is the ability of the central nervous system to assess the degree of reliance on primary sensory feedback modalities for postural control (9). The influence that each analyzer provides to the brain varies with the complexity of the motor task, environmental conditions, and fidelity of external stimuli (6). For example, the brain relies on cutaneous and kinesthetic analyzers when standing in an unperturbed and quiet condition, while during perturbed standing on an unstable surface, the brain has to increase the weight of other sensory modalities that are more reliable, such as visual analyzer (10).

The process of adjusting the sensory contributions for balance control is defined as sensory reweighting (6). This reweighting provides a compensatory mechanism for analyzing altered afferent stimuli in situations such as musculoskeletal injuries or aging (11–14).

Strobe glasses are a modern technology that reduces visual information and forces sensory reweighting to other analyzers within multisensory integration (10). Strobe glasses consist of liquid crystal lenses that oscillate between opacity and transparency over defined periods of time (15). Strobe training is a form of visual training where the athlete has to practice a motor task during intermittently dark conditions (16). In theory, strobe training improves visual, perceptual, and cognitive skills, which will lead to enhanced sporting performance (16). By changing the visual perception in an intermittent, repeated, and fast manner, the athlete is forced either to use more effectively the limited visual stimuli that he receives or to use the information that comes from other analyzers (the kinesthetic analyzer or the auditory analyzer) (16). Moreover, realizing the motor task under more difficult conditions because of the visual training will change the subjective perspective toward the motor task made under normal conditions (17). The premise of strobe training is that practicing motor tasks during stroboscopic vision will encourage visual-cognitive processes to adapt to cope with the suboptimal information available (18).

Strobe training was used in different sports disciplines, such as badminton (19), baseball (4, 20), cricket (21), football (22), ice hockey (17, 23), tennis (24, 25), volleyball (26), and softball (27).

In theory, strobe training may influence some perceptual and cognitive abilities (28). Some of the benefits of strobe training suggested by the literature are: improved hitting accuracy (24), better visual-spatial memory (18), better short-term visual memory (29), higher decision accuracy (21), better anticipatory timing (17), more efficient motion coherence and higher attention in central vision (28), and improved reactive agility (26). The most important benefit is considered to be the transfer to better sports skill performance (21–23, 25).

When talking about strobe training protocols, the main advantage is that the training session can take place in the natural context of the sport. On the other hand, this variability of the training environment leads to a high variability of the training protocol. This variability manifests in three forms: first, about the length (of the whole intervention with the strobe glasses, of every session, of the period of time when the athlete wears the glasses per session); second, about the motor tasks used in the session (whether to use sport-specific tasks or general fitness tasks); and third, about the mode and level of using the glasses (16). In most of the studies that applied strobe technology, researchers used sport-specific tasks using a similar protocol to the normal session without glasses (16).

Coaches should avoid using strobe training with athletes who experience epilepsy or epileptic seizures (16). Moreover, because athletes have to perform while their vision is impaired, they should perform motor tasks below their performance level for safety measures: with lower speed or with protection (16).

The benefits of strobe training may be transferred to other sports, such as climbing (30). The first argument for applying this training method to climbing is sensory reweighting: the climber will be forced to process more efficiently the external cutaneous stimuli that come from the holds and also the internal kinesthetic and proprioceptive stimuli that come from muscles and tendons. This improved focus on holds and proprioception may lead to increased body awareness and higher movement efficacy on the wall. The second argument for possible benefits in climbing comes from the increased central and peripheral visual acuity and better visual memory that can lead to better calibration of the hand grip and improved attention toward foot holds. Another possible benefit of climbing would be for realizing a better visualization before climbing: the lack of visual stimulus forces the athlete to start the route with a very clear ascent plan, because his ability to adapt during the ascent is limited by the fatigue of the additional visual effort. Lastly, because strobe training increases anticipatory timing and decision accuracy, it may have benefits on the tactical thinking of a climber (31).

In a recent study, Vasile and Stănescu (15) applied strobe technology to nine advanced youth climbers. The climbers performed six training sessions wearing strobe glasses and after every session a feedback questionnaire was applied to run a thematic analysis. Their research question was “How do strobe glasses influence climbing technique?”; therefore, they asked climbers about: how they adapted to climbing wearing strobe glasses; which were the benefits of strobe training in climbing; and the disadvantages of climbing with the glasses on. Similar to the study conducted by Wilkins et al. (22), the athletes found climbing wearing strobe glasses challenging, interesting, fun but also demanding. The thematic analysis concluded that strobe training in climbing would have benefits on focused and distributive attention, memory, visualization, optimal ascending speed, body placement on the wall, sensory reweighting toward proprioception, central and peripheral vision, and coordination. The disadvantages of climbing with the glasses on were mental fatigue, dizziness, and the inability to distinguish the colors of some holds. This study was the first study that implemented strobe training in youth climbing and highlighted some methodological guidelines about applying strobe glasses in climbing (15). The authors used two pairs of glasses on levels 1 and 2, on mode A with both lenses blinking simultaneously. The athletes performed similar training sessions to their normal climbing session in the bouldering gym, in the lead gym, on the Spraywall, and on the MoonBoard with a session that lasted around 2 h. The intensity of the climbed routes was lower than each climber's level of performance, for safety. The strobe training session was an additional climbing session that the climbers did weekly.

Having in mind the benefits of strobe training suggested in other sports and in climbing, we hypothesized that strobe training can influence multiple training components: psychological training (by increasing attention, memory, visual memory, and cognitive agility); and tactical training (by increasing visualization, decisional capacity, reasoning, processing speed, choosing the optimal ascending speed, spatial skills, and reactivity). By performing climbing tasks with the glasses on during an extra training session, the volume of training will increase and can sustain the overall preparation of a climber. The increased training volume will enhance physical and technical training and can lead to enhanced performance.

Our first research question was whether strobe training can increase climbing performance. Our second research question was which are the training components that are influenced by strobe training. To this end, we proposed a strobe training program composed of 20 additional climbing sessions and highlighted how much the climbing performance increased in the experimental group in comparison to the control group.

The aim of the research was to determine the efficiency of a program composed of 20 additional climbing sessions with strobe glasses. This program was meant to examine the increase in climbing performance, by developing cognitive skills (spatial skills and reactivity), motor-cognitive skills (cognitive agility, visual memory, visual processing speed), and climbing-specific parameters.

## 2 Materials and methods

### 2.1 Participants

The participants were recruited during a National Championship event, where the main investigator explained the purpose of the study, the main objectives, and the intervention to the climbers, their coaches, and their parents (March 2021). The study was conducted on 17 youth elite climbers (10 male, 7 female; age range 13–20 years) from Romania (*M* = 16.59; DS = 2.00), representing the entire National Youth Climbing Team.

The inclusion criteria were as follows: age 13–20 years (so that they would participate in youth competitions); a minimum of three climbing sessions per week; a minimum climbed grade of 7a (on-sight or red-point, bouldering or lead, on artificial walls or on rocks, performance realized in the last year before enrolling into the study); and participation in National/International competitions (each climber participated in at least one National competition in the last year before enrolling into the study). According to Draper et al. (32), we analyzed advanced climbers, but the participants represented the entire National Youth Climbing Team, so we considered them as being a representative sample for elite climbers.

Their red-point performance varied from 7a to 9a, while their on-sight performance varied from 6c to 8b. Their climbing experience was in the range of 1–12 years (*M* = 6.94; DS = 3.01). The athletes had a number of climbing sessions that were in the range of 3–7 per week (*M* = 4.29; DS = 1.31). The length of a climbing session was in the range of 2–4 h (*M* = 2.85; DS = 0.52).

The research group was divided into a control group (eight climbers) and an experimental group (nine climbers) by selective distribution. In terms of differences by gender, the experimental group was formed by nine climbers (five male, four female). The control group was formed by eight climbers (five male, three female). We selected the climbers from the capital of Romania, Bucharest, to be in the experimental group and the ones from other cities to be in the control group, because the strobe training sessions were to be conducted in Bucharest. Moreover, because we used selective distribution into the groups, we tried to match every control participant to an experimental participant according to their level of performance and climbing experience.

### 2.2 Instruments

The first instrument was an introductive questionnaire, which was sent online to each participant. The collected variables were age, gender, experience, number of sessions per week, on-sight performance, and red-point performance. Climbing performance was reported on French scale and converted into Watts' scale (32).

The second instrument was the Cognitrom battery, which was completed in a laboratory setting by each participant (33). The evaluation lasted approximately 1 h and the climber had to complete the tests on the computer only with the investigator by his side to give instructions for the test. The license for applying this battery belongs to one of the researchers. This technology was used because it is one of the cognitive skills evaluation technologies previously performed on the Romanian population.^1^

The Cognitrom battery evaluated cognitive skills according to its methodology: spatial skills (mental image transformation, spatial orientation, and image generation) and reactivity skills (simple reaction time, choice reaction time, and memory access reaction time). The mental image transformation test supposed that the participant identified a specific geometric form that was identical to the first seen but in a rotated way. The spatial orientation test supposed that the participant identified the same geometric figure as the one first seen but from another angle. The image generation test showed that the participant identified the correct image composed of the superimposing of the two images that they saw previously. The simple reaction time test illustrated that the participant had to press the space bar when the visual stimulus appeared. The choice reaction time test determined that the participant had to press a certain button on the keyboard if two geometrical figures that they saw on the screen were close to each other and another certain button on the keyboard if the two geometrical figures were not next to each other. The memory access reaction time test showed that the participant identified if the sixth letter that appeared on the screen was one of the first five letters that appeared on the screen previously (33).

The third instrument was the Witty SEM system. The apparatus belongs to the “Alexandru Partheniu” Interdisciplinary Research Center from the National University of Physical Education and Sports and the license for applying this system belongs to the research team from the University. Witty SEM technology is a computerized apparatus that evaluates and trains several visual, cognitive, and sensory-motor skills, along with Senaptec Sensory Station, Sports Vision Performance, and Visual Edge Performance Trainer (34). The Witty SEM technology is used for evaluation and specific training for reactivity, agility, and coordination.^2^ It has the advantage that the researcher can add the motor task in addition to the evaluation of cognitive skills, naming them motor-cognitive skills. Some of the motor-cognitive skills that can be evaluated with Witty SEM technology are attention, brain speed, intelligence, cognitive agility, visual processing speed, and visual memory.

**Figure 1:**
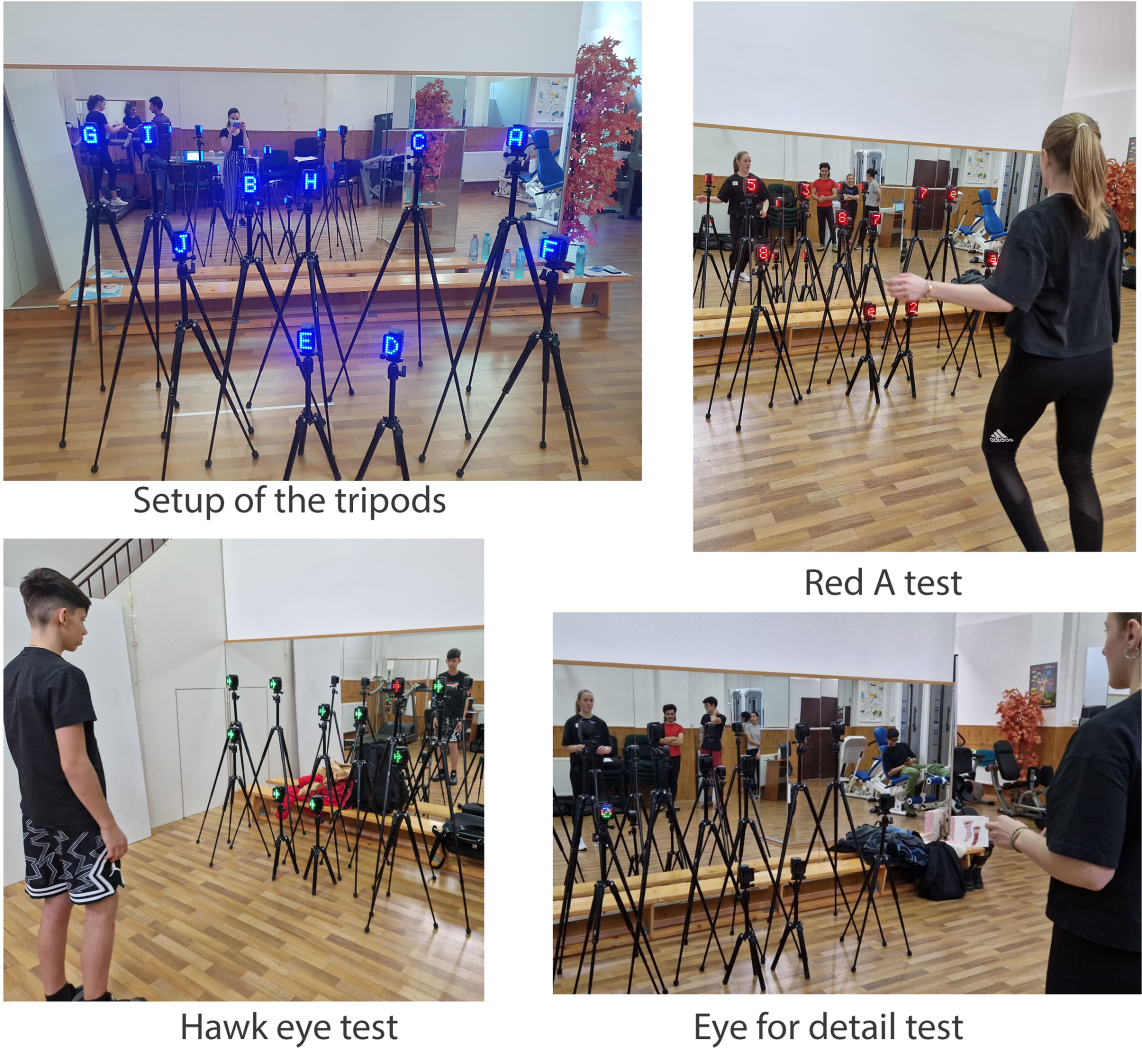
Witty SEM instrument and tests.

The Witty SEM system is composed of 10 tripods that were arranged in a position that resembled a bouldering problem (four starting points and six more tripods that resembled future holds that the climber had to reach). The Witty SEM system evaluated motor-cognitive skills according to its methodology: cognitive agility; visual processing speed (time for visual processing and a number of visual processing errors); and visual memory (time for visual memory and a number of visual memory errors) (33). The cognitive agility was measured with the red A test. On the 10 tripods appeared 10 red stimuli and the participant had to touch the tripod that showed the red letter “a.” The output was the number of seconds for completing the task. The visual processing speed was measured with the hawk eye test. On the 10 tripods appeared 9 green stimuli and the participant had to touch the 10th tripod that showed the red stimulus. The output was: the number of seconds for completing the task and the number of errors in completing the task. The visual memory was measured with the eye for detail test. On the 10 tripods, 3 visual stimuli were shown, 2 of them being identical. The participant had to touch the tripods that were similar. The output was the number of seconds for completing the task and the number of errors in completing the task (33) (Figure 1).

The fourth instrument was the International Rock Climbing Research Association (IRCRA) performance-related test battery for climbers but adapted to the existing equipment from the climbing gym.^3^ We also added three more tests. We used the battery for evaluating climbing-specific parameters:

a. Climbing-specific foot raise with rotation (measured at the espalier) (left foot and right foot and the used variable was the mean of left and right) (after one repetition, measured by the height of foot in cm).
b. Climbing-specific foot raise without rotation (measured at the espalier) (left foot and right foot and the used variable was the mean of left and right) (after one repetition, measured by the height of foot in cm).
c. Finger hang (measured at the Metolius board on slopers, medium edge, and small edge and the used variable was the mean of the three positions) (after one repetition, measured by time to failure in seconds).
d. Power slap (measured at the Gullich board) (measured by slap height in cm).
e. Bent arm hang (measured with both arms and then with each arm and the used variables were for the exercise with both arms and the mean for left and right arm) (measured by time in seconds).
f. Pull-up shoulder endurance (measured at the Metolius board on slopers, medium edge, and small edge and the used variable was the mean of the three positions) (measured by the number of repetitions to fatigue).
g. Plank (measured by time in seconds to fatigue after one repetition).
h. 90° bent leg raise (measured by time in seconds to fatigue after one repetition).
i. Core strength (added test: maximal number of crunches from the starting position of hanging at the fixed bar).
j. Push-up shoulder endurance (added test) (measured by number of repetitions to fatigue).
k. Dips (added test) (measured by number of repetitions to fatigue).

### 2.3 Testing procedure

The study had an initial testing (T1) (July–October 2021), an intervention period (January 2022–December 2022), and a final testing (T2) (December 2022–January 2023). The intervention lasted a calendar year.

The first phase of the testing was sending the online introductory questionnaire. The second phase of the testing was applying the Cognitrom battery, Witty SEM system, and the IRCRA performance-related test battery for climbers. The Cognitrom battery assessment was on the computer and the climbers started with the reactivity skills tests and then with the spatial skills tests. The Witty SEM assessment was in the University laboratory and was conducted by their researcher: the first applied test was for measuring cognitive agility, then for measuring visual processing speed, and then for measuring visual memory. For the IRCRA performance-related test battery for climbers, the evaluation took place following the test manual's instructions.

For the control group, given the fact that they were from different cities, the evaluation took place over three consecutive days: day 1 for the Witty SEM evaluation; day 2 for the Cognitrom assessment; and day 3 for the climbing-specific evaluation. The climbers from the control group were divided by the city they came from; thus each evaluation took place with the investigator and two or three climbers. For the intervention group, the evaluation took place over three separate days, depending on their personal schedule and their training schedule (1 day for each instrument). The motor evaluation took place after at least 1 rest day after a climbing training session.

### 2.4 Intervention

The control group and the intervention group continued the normal climbing sessions that they did with their principal trainer. Therefore, both groups continued their normal practice of 3–7 weekly sessions. Both groups had the same competitive goals and the same periodization on macrocycle, all of them being part of the National climbing team and participating in the same competitions. Because of that, the intensity and volume of their normal climbing sessions were considered similar, having the same increasing and decreasing of the training parameters depending on the moment during the macrocycle. In addition, the fact that the performance, experience, number of training sessions, and climbing-specific parameters did not statistically differ between the groups was another argument for implementing the intervention.

**Figure 2:**
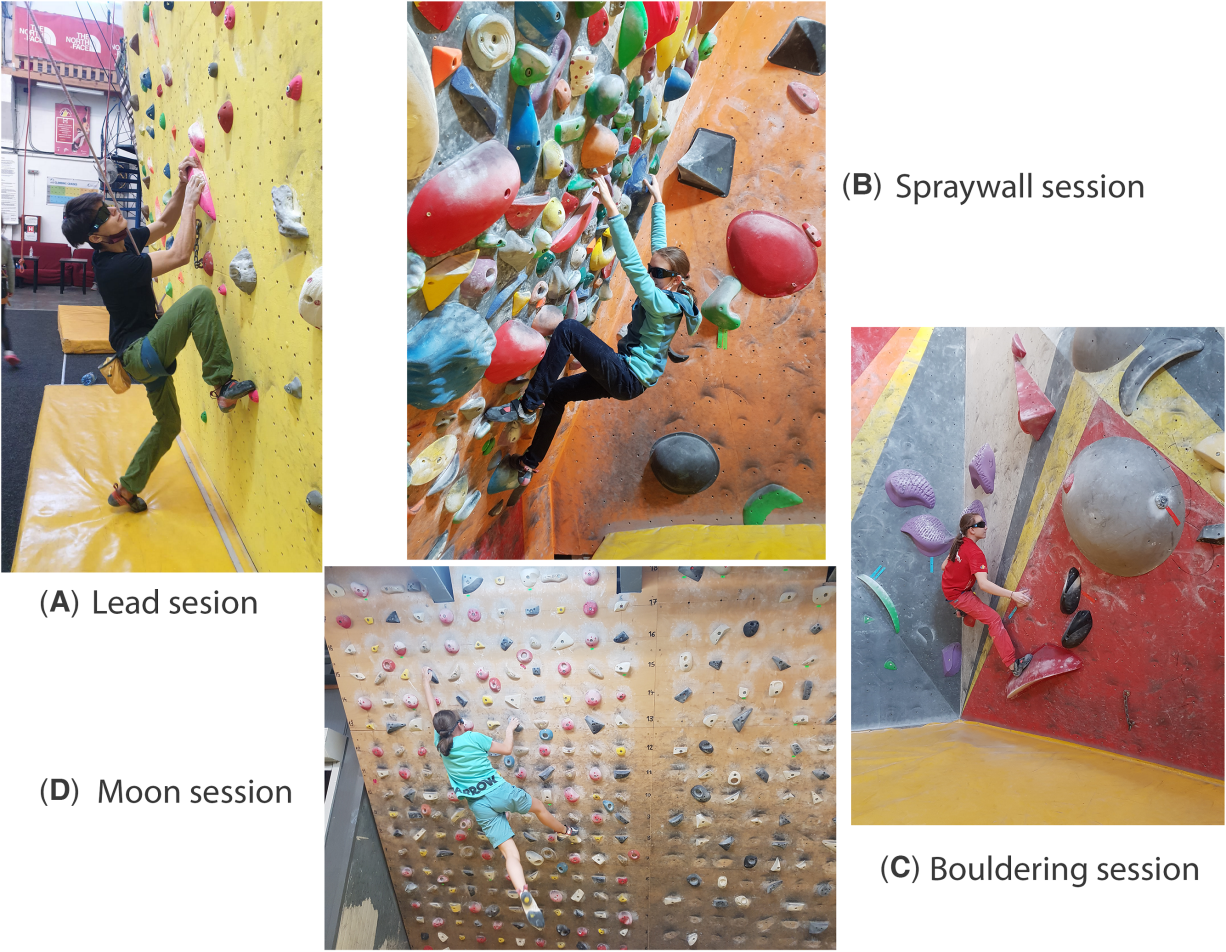
Strobe training sessions.

The training intervention consisted of sessions that were similar to the normal climbing sessions without the glasses. Thus, the athletes climbed with the glasses on their normal training walls: on the bouldering wall, on the lead wall, on the MoonBoard, and on the Spraywall (Figure 2). During the bouldering and lead sessions, climbers did approximately 8–10 routes with grades depending on periodization. During the MoonBoard sessions, climbers did routes on the standardized moon board, with routes chosen from the standardized digital application (approximately 8–10 routes per session). During Spraywall sessions, climbers had to do routes on a board with multiple holds that did not resemble any specific route. The investigator conceived routes (approximately five routes with 20–30 moves).

Every session lasted approximately 2 h. The climbers had 20 strobe training sessions that took place during one calendar year. The strobe session was additional to their climbing schedule done with their principal trainer. The strobe session was once a week, depending on the periodization that the climber had with their principal trainer. In the preparatory period, the climbers had three strobe sessions per month, having to climb on all surfaces from the climbing gym alternatively, with an intensity of 70%–80%. In the competitive period, the climbers had one strobe session per month, with an intensity of 85%–95%. In the competitive period, the objective for every session was chosen according to the next competition (for example, if they had a bouldering competition during that month, they had a strobe session at the bouldering wall). In the transition period, the climbers had one to two strobe sessions per month, having to climb on all surfaces from the climbing gym alternatively, with an intensity of 40%–50%. The intensity of the session was determined by the grades of the routes chosen by the investigator to climb in every session, as a percentage reported to every climber`s maximum performance from the experimental group.

For security reasons, during the bouldering sessions, the climbers had to declimb the routes. During the lead sessions, we used only top-roping. In addition, the routes chosen to climb were a grade lower than the maximum performance of the climbers.

### 2.5 Statistical analysis

The statistical analysis was performed using SPSS software with descriptive statistics, paired Student *t*-tests, and Wilcoxon tests.

## 3 Results

First, we used the independent *t*-test to check that there were no differences between the control group and the experimental group in terms of experience and performance. The analysis explained that there were no differences between the groups in terms of on-sight performance (*p* = 0.882), red-point performance (*p* = 0.515), experience (*p* = 0.814), and number of climbing sessions per week (*p* = 0.478).

Second, we used the independent *t*-test to check that there were no differences between the control group and the experimental group in terms of climbing-specific variables after initial testing (T1). The analysis explained that there were no differences between the control group and experimental group for all the climbing-specific parameters: foot raise with rotation right (*p* = 0.156), foot raise with rotation left (*p* = 0.219), foot raise without rotation right (*p* = 0.549), foot raise without rotation left (*p* = 0.261), finger hang slopers (*p* = 0.602), finger hang medium edge (*p* = 0.366), finger hang small edge (*p* = 0.730); Gullich power slap left (*p* = 0.370), Gullich power slap right (*p* = 0.289), bent arm hang both arms (*p* = 0.723), bent arm hang left (*p* = 0.886), bent arm hang right (*p* = 0.901), pull-ups slopers (*p* = 0.623), pull-ups medium edge (*p* = 0.709), pull-ups small edge (*p* = 0.590), plank (*p* = 0.557), 90° bent leg raise (*p* = 0.306), push-ups (*p* = 0.517), core strength (*p* = 0.226), and dips (*p* = 0.125).

The first research question was to examine if the strobe training intervention can improve climbing performance. To test this hypothesis (H1), we used the paired *t*-test to see if there were any differences between climbing performance (on-sight and red-point) at T1 versus T2.

The second research question was to examine if strobe training intervention can improve cognitive performance (H3), motor-cognitive performance (H5), and climbing-specific parameters (H6). To test these hypotheses, we used paired *t*-tests. H2 tested if climbing experience can influence the effect that the intervention had on increasing climbing performance, using the Wilcoxon test. H4 tested if age can influence the effect that the intervention had on increasing cognitive performance, using the Wilcoxon test.

### 3.1 H1: strobe training intervention will improve on-sight performance and red-point performance

To test H1, we used the paired *t*-test for both the control and experimental groups, to see if there were any differences between T1 and T2 for climbing performance (on-sight and red-point).

In Table 1, we can see the descriptive analysis for on-sight performance and red-point performance and the results from the paired *t*-test.

**Table 1:** *t-*test for on-sight performance and red-point performance for control group and experimental group.

		Mean	Standard deviation	Paired *t-*test (T1 - T2)	Sig (two-tailed)	Cohen D effect size
Control group	On-sight performance T1	3.281	0.573	−2.646	0.033	0.21
	On-sight performance T2	3.406	0.565			
	Red-point performance T1	4.187	0.678	−0.552	0.598	—
	Red-point performance T2	4.218	0.646			
Experimental group	On-sight performance T1	3.222	0.995	−5.965	0.000	0.38
	On-sight performance T2	3.583	0.892			
	Red-point performance T1	3.888	1.092	−5.093	0.001	0.36
	Red-point performance T2	4.257	0.979			

From Table 1, we see that for the control group, the on-sight performance increased [*t* (7) = 2.646, *p* = 0.033], but with a small effect size (*d* = 0.21). For the experimental group, the on-sight performance increased [*t* (8) = 5.965, *p* = 0.000], with a small effect size (*d* = 0.38), and the red-point performance increased [*t* (8) = 5.093, *p* = 0.001], with a small effect size (*d* = 0.36).

In other words, for the experimental group, we objectified significant differences between tests for both types of performances, with small effect sizes.

### 3.2 H2: climbing experience influenced the effect of strobe training intervention on performance

The climbers had an experience that varied between 1 and 12 years (*M* = 6.94; SD = 3.01). For testing the second hypothesis, we divided the research group into four subgroups, depending on climbing experience (control group with climbing experience less than 7 years, control group with climbing experience over 7 years, experimental group with climbing experience less than 7 years and experimental group with climbing experience over 7 years).

To test H2, we used the Wilcoxon test for both the control and experimental groups, divided by subgroups to see if there were any differences between T1 and T2 for climbing performance (on-sight and red-point).

In Table 2, we can see the descriptive analysis performance by subgroups and the results from the Wilcoxon test.

**Table 2:** Wilcoxon test for performance by subgroups divided by experience.

		Mean	Standard deviation	Z Wilcoxon test (T1 − T2)	Sig (two-tailed)	Effect size
Control group with less experience (five climbers)	On-sight performance T1	3.10	0.65	−1.173	0.083	—
	On-sight performance T2	3.25	0.68			
	Red-point performance T1	3.95	0.75	—	—	—
	Red-point performance T2	3.95	0.64			
Control group with more experience (three climbers)	On-sight performance T1	3.583	0.288	−1.000	0.317	—
	On-sight performance T2	3.666	0.144			
	Red-point performance T1	4.583	0.288	−1.000	0.317	—
	Red-point performance T2	4.666	0.381			
Experimental group with less experience (four climbers)	On-sight performance T1	2.437	0.239	−1.841	0.066	0.92
	On-sight performance T2	2.937	0.125			
	Red-point performance T1	3.062	0.314	−1.841	0.066	0.92
	Red-point performance T2	3.500	0.456			
Experimental group with more experience (five climbers)	On-sight performance T1	3.850	0.911	−2.236	0.025	0.99
	On-sight performance T2	4.100	0.911			
	Red-point performance T1	4.550	1.036	−1.841	0.066	0.82
	Red-point performance T2	4.864	0.854			

From Table 2, we can see that there are almost significant differences for the experimental group indifferent to the experience level (*p* = 0.066 for the subgroup with less experience for both on-sight performance and red-point performance, *p* = 0.066 for the subgroup with more experience for red-point performance, and *p* = 0.025 for the subgroup with more experience for on-sight performance).

In other words, the intervention with strobe glasses would have benefits for performance indifferent to the climbing experience but would have the best benefits for the climbers with more experience.

### 3.3 H3: strobe training intervention will improve spatial skills (mental image transformation, image generation, and spatial orientation) and reactivity (simple reaction time, choice reaction time, and memory access reaction time)

To test H3, we used the paired *t*-test for both the control and experimental groups to see if there were any differences between T1 and T2 for cognitive performance variables (mental image transformation, image generation and spatial orientation, simple reaction time, choice reaction time, and memory access reaction time).

In Table 3, we can see the descriptive analysis for spatial skills, reactivity variables, and the results from the paired *t-*test.

**Table 3:** *t-*test for spatial skills and reactivity variables for control group and experimental group.

		Mean	Standard deviation	Paired *t-*test (T1−T2)	Sig (two-tailed)	Cohen D effect size
Control group	Mental images transformation T1	14.38	2.774	−0.919	0.388	0.22
	Mental images transformation T2	15.00	2.828			
	Spatial orientation T1	16.63	1.996	0.243	0.815	0.05
	Spatial orientation T2	16.50	2.878			
	Image generation T1	11.00	2.138	−0.284	0.785	0.14
	Image generation T2	11.25	1.282			
	Simple reaction time T1	254.00	27.077	0.708	0.502	0.38
	Simple reaction time T2	243.25	30.523			
	Choice reaction time T1	950.38	274.688	−0.749	0.478	0.29
	Choice reaction time T2	1,026.00	247.298			
	Memory access reaction time T1	1,049.13	181.259	0.673	0.523	0.09
	Memory access reaction time T2	1,031.13	182.143			
Experimental group	Mental images transformation T1	13.00	2.550	−5.124	0.001	1.27
	Mental images transformation T2	15.89	1.965			
	Spatial orientation T1	14.78	1.787	−4.899	0.001	1.14
	Spatial orientation T2	16.78	1.716			
	Image generation T1	10.33	2.179	−9.141	0.000	1.59
	Image generation T2	13.78	2.167			
	Simple reaction time T1	291.44	68.737	3.007	0.017	0.99
	Simple reaction time T2	240.22	23.679			
	Choice reaction time T1	1,142.44	337.453	1.225	0.255	0.16
	Choice reaction time T2	1,089.22	321.932			
	Memory access reaction time T1	1,148.78	497.315	2.280	0.052	0.23
	Memory access reaction time T2	1,043.33	370.857			

From Table 3, we see that for the control group, there are no significant differences between the first testing and the second testing (*p* > 0.05). For the experimental group, from the spatial skills, mental image transformation variable increased [*t* (8) = 5.124, *p* = 0.001], with a large effect size (*d* = 1.27); spatial orientation increased [*t* (8) = 4.899, *p* = 0.001], with a large effect size (*d* = 1.14); image generation increased [*t* (8) = 9.141, *p* = 0.000], with a large effect size (*d* = 1.59). For the experimental group, from the reactivity skills, simple time reaction improved [*t* (8) = 3.007, *p* = 0.017], with a large effect size (*d* = 0.99); there were no significant differences for choice reaction time and memory access reaction time.

In other words, for the experimental group, we objectified significant differences between tests for the majority of cognitive variables (mental image transformation, spatial orientation, image generation, and simple reaction time), with large effect sizes.

### 3.4 H4: age influenced the effect of strobe training intervention on cognitive variables (mental image transformation, spatial orientation, image generation, simple reaction time, and memory access reaction time)

To test the fourth hypothesis, we divided the research group into four subgroups, depending on age (above and below 16 years, according to the International Federation of Sport Climbing (IFSC) age limit for competing in Senior competitions). Thus, the control group aged under 16 years was formed by two climbers, the control group aged above 16 years was formed by six climbers, the experimental group aged under 16 years was formed by seven climbers, and the experimental group aged above 16 years was formed by two climbers.

To test H4, we used the Wilcoxon test for both the control and experimental groups, divided by subgroups to see if there were any differences between T1 and T2 for cognitive performance variables (mental image transformation, spatial orientation, image generation, simple reaction time, and memory access reaction time).

In Table 4, we can see the significant results from the Wilcoxon test only for the experimental group aged under 16 years. For the other subgroups, there were no significant differences between tests.

**Table 4:** Wilcoxon test for spatial skills and reactivity variables for the experimental group below 16 years old.

		Z Wilcoxon test (T1 − T2)	Sig (two-tailed)	Effect size
Experimental group below 16 years old	Mental image transformation	−2.388	0.017	0.81
	Spatial orientation	−2.214	0.027	0.70
	Image generation	−2.379	0.017	0.81
	Simple reaction time	−2.366	0.018	0.80
	Memory access reaction time	−2.366	0.018	0.80

In other words, the strobe training intervention would have greater benefits on cognitive skills only for climbers aged under 16 years.

### 3.5 H5: strobe training intervention will improve motor-cognitive skills (cognitive agility, visual processing speed, and visual memory)

To test H5, we used the paired *t*-test for both the control and experimental groups, to see if there were any differences between T1 and T2 for motor-cognitive performance variables (cognitive agility, visual processing speed, and visual memory).

In Table 5, we can see the descriptive analysis for spatial skills, reactivity variables, and the results from the paired *t*-test.

**Table 5:** *t-*test for motor-cognitive variables for control group and experimental group.

		Mean	Standard deviation	Paired *t-*test (T1 − T2)	Sig (two-tailed)	Cohen D effect size
Control group	Visual memory T1	0.265	0.0860	−1.033	0.336	0.46
	Visual memory T2	0.323	0.1677			
	Visual memory errors T1	2.50	0.756	1.528	0.170	0.34
	Visual memory errors T2	2.25	0.707			
	Visual processing T1	0.049	0.0156	1.453	0.189	0.28
	Visual processing T2	0.045	0.0121			
	Visual processing errors T1	3.38	1.408	1.158	0.285	0.28
	Visual processing errors T2	3.00	1.309			
	Cognitive agility T1	57.674	4.945	2.664	0.032	0.24
	Cognitive agility T2	56.524	4.814			
Experimental group	Visual memory T1	0.300	0.067	3.728	0.006	0.16
	Visual memory T2	0.185	0.074			
	Visual memory errors T1	2.67	0.707	5.500	0.001	1.96
	Visual memory errors T2	1.44	0.527			
	Visual processing T1	0.045	0.012	3.561	0.007	1.39
	Visual processing T2	0.033	0.007			
	Visual processing errors T1	4.33	1.00	8.000	0.000	1.94
	Visual processing errors T2	2.11	1.269			
	Cognitive agility T1	62.688	10.876	5.393	0.001	1.30
	Cognitive agility T2	51.484	5.480			

From Table 5, we see that for the control group, there is only one motor-cognitive variable that increased: cognitive agility [*t* (7) = 2.664, *p* = 0.032], with a small effect size (*d* = 0.24). For the experimental group, visual memory increased [*t* (8) = 3.728, *p* = 0.006], with a very small effect size (*d* = 0.16); number of visual memory errors decreased [*t* (8) = 5.500, *p* = 0.001], with a large effect size (*d* = 1.96); visual processing skill increased [*t* (8) = 3.561, *p* = 0.000], with a large effect size (*d* = 1.39); number of visual processing errors decreased [*t* (8) = 8.000, *p* = 0.000], with a large effect size (*d* = 1.94); and cognitive agility increased [*t* (8) = 5.393, *p* = 0.001], with a large effect size (*d* = 1.30).

In other words, for the experimental group, we objectified significant differences between tests for all motor-cognitive variables (cognitive agility, visual processing speed, and visual memory), with large effect sizes.

### 3.6 H6: strobe training intervention will improve climbing-specific parameters

To test H6, we used the paired *t*-test for both the control and experimental groups, to see if there were any differences between T1 and T2 for climbing-specific parameters (foot raise with rotation, foot raise without rotation, finger hang, pull-ups, push-ups, dips, core strength).

In Table 6, we can see the descriptive analysis for the climbing-specific variables and the results from the paired *t*-test.

**Table 6:** *t-*test for climbing-specific parameters for control group and experimental group.

		Mean	Standard deviation	Paired *t-*test (T1 − T2)	Sig (two-tailed)	Cohen D effect size
Control group	Foot raise with rotation T1	164.313	19.735	−1.418	0.199	0.49
	Foot raise with rotation T2	172.250	11.862			
	Foot raise without rotation T1	157.125	20.852	−2.640	0.033	0.72
	Foot raise without rotation T2	170.438	15.425			
	Finger hang on medium edge T1	58.13	15.385	0.445	0.670	0.12
	Finger hang on medium edge T2	56.25	17.540			
	Finger hang on small edge T1	43.25	16.663	0.000	1.000	0.00
	Finger hang on small edge T2	43.25	18.156			
	Pull-ups on slopers T1	16.88	6.105	−0.080	0.939	0.01
	Pull-ups on slopers T2	17.00	7.616			
	Pull-ups on medium edge T1	12.13	5.693	−1.454	0.189	0.31
	Pull-ups on medium edge T2	14.13	7.060			
	Pull-ups on small edge T1	8.38	4.406	−2.072	0.077	0.45
	Pull-ups on small edge T2	11.00	7.071			
	Push-ups T1	29.38	13.169	2.198	0.064	0.13
	Push-ups T2	27.63	13.617			
	Dips T1	10.50	6.949	0.000	1.000	0.00
	Dips T2	10.50	8.896			
	Core strength T1	96.88	16.234	2.620	0.034	0.68
	Core strength T2	81.88	26.589			
Experimental group	Foot raise with rotation T1	175.444	12.531	−4.213	0.003	0.47
	Foot raise with rotation T2	181.000	12.886			
	Foot raise without rotation T1	145.778	29.975	−2.570	0.033	0.49
	Foot raise without rotation T2	160.167	30.344			
	Finger hang on medium edge T1	51.11	15.584	−2.548	0.034	0.64
	Finger hang on medium edge T2	62.56	20.342			
	Finger hang on small edge T1	40.33	17.371	−3.339	0.010	0.64
	Finger hang on small edge T2	51.89	18.738			
	Pull-ups on slopers T1	15.22	7.328	−1.367	0.209	0.18
	Pull-ups on slopers T2	16.44	6.616			
	Pull-ups on medium edge T1	11.11	5.278	−1.976	0.084	0.25
	Pull-ups on medium edge T2	12.33	4.637			
	Pull-ups on small edge T1	7.33	3.391	−2.159	0.063	0.45
	Pull-ups on small edge T2	8.78	3.073			
	Push-ups T1	33.00	9.247	−4.102	0.003	0.74
	Push-ups T2	40.56	11.182			
	Dips T1	5.56	5.593	−3.931	0.004	0.52
	Dips T2	8.44	5.411			
	Core strength T1	82.56	28.125	−1.028	0.334	0.29
	Core strength T2	91.56	33.444			

From Table 6, we see that for the control group, there is only one climbing-specific variable that increased: foot raise without rotation [*t* (7) = 2.640, *p* = 0.033], with a medium effect size (*d* = 0.72). For the experimental group, foot raise with rotation increased [*t* (8) = 4.213, *p* = 0.003], with a small effect size (*d* = 0.44); foot raise without rotation increased [*t* (8) = 2.570, *p* = 0.033], with a small effect size (*d* = 0.47); finger hang on medium edge increased [*t* (8) = 2.548, *p* = 0.034], with a medium effect size (*d* = 0.63); finger hang on small edge increased [*t* (8) = 3.339, *p* = 0.010], with a medium effect size (*d* = 0.64); push-up shoulder endurance increased [*t* (8) = 4.102, *p* = 0.003], with a medium effect size (*d* = 0.73); dips increased [*t* (8) = 3.931, *p* = 0.004], with a medium effect size (*d* = 0.50).

In other words, for the control group, we objectified significant differences only for a variable that measured flexibility, whereas for the experimental group, we objectified significant differences between tests for many climbing-specific variables: mainly for flexibility (foot raise with rotation and without rotation) and upper body strength (finger hang, push-up shoulder endurance, and dips), but with small and medium effect sizes.

## 4 Discussion

The present study detailed a training program using strobe glasses as a form of visual training for enhancing performance in youth elite climbing. The objective of the strobe training sessions was to develop cognitive skills and motor-cognitive skills, as part of the cognitive training of an athlete. The program consisted of 20 additional training sessions organized once a week depending on the periodization by macrocycle, during one calendar year. Regarding which are the training components that are influenced by strobe training in climbing, we demonstrated benefits for climbing performance (on-sight and red-point), cognitive performance (spatial skills and reactivity), motor-cognitive performance (cognitive agility, visual processing speed, and visual memory) and climbing-specific parameters.

Regarding climbing performance, we demonstrated that strobe training improves both types of performances (on-sight performance and red-point performance), but with small effect sizes. This result suggests that strobe training improves both recreational climbing and competitive climbing, as increasing performance on the first try, but also after multiple tries. This highlights that strobe training can be used as a training method both for climbers who have a competitive goal (where most climbing is on-sight or flash), but also for climbers who have a personal goal (realizing a personal best). Moreover, the result that the performance was enhanced with a small effect size means that strobe training should be a method used at an elite level, as it does not have large benefits in terms of athletic performance (from a physical or technical point of view). Strobe training should be used for enhancing other training components (such as cognitive training, tactical training, or psychological training), which will then lead to enhanced athletic performance. The same idea was highlighted from the result that strobe training had better benefits for the climbers with more experience, in comparison to those with less experience. In fact, a Canadian stage-based approach for climber development explained that visual training that enhances visual acuity should be developed during the seventh stage, at the age level of 18+ years, where the main goal is winning for a living.^4^

Regarding cognitive performance, we demonstrated that strobe training improves some cognitive skills, more specifically spatial skills (mental image transformation, spatial orientation, and image generation) and simple reaction time, with large effect sizes. Moreover, we demonstrated that regarding cognitive skills, strobe training acts more on spatial skills, rather than on reactivity skills. Spatial skills are crucial for elite climbers, in order for them to properly control their center of mass during the act of climbing (35). Climbing spatial orientation leads to the minimization of jerky movements and to more smoothly linked moves, which will lead to more efficient climbing (36). Moreover, spatial analysis is an important skill for elite climbers, as rapid improvement in climbing performance is believed to be influenced by the rapidly adapting visual-motor system (37). Development of the visual-motor system occurs over longer periods of time, such as months or years (38). This is where strobe training acts upon, developing the visual-motor coordination system. Regarding reactivity skills, lead climbing and bouldering climbing are not disciplines performed under time pressure, but a high reaction time in terms of automatic activation of grasping actions is an important ability for a climber's performance (39). Reaction time is trained especially for speed climbing (40). Our result suggested that strobe training can enhance reactivity skills in climbing, which can benefit in all subdisciplines: speed climbing, bouldering, or lead climbing. Another interesting result from developing cognitive performance was that strobe training has greater benefits for climbers aged under 16 years. This result is in accordance with previous studies that stated that youth athletes rely more on visual input than adults (41). The importance of cognitive training in climbing is supported by previous research, which highlighted the need for enhanced visualization (42), improved cognition (43), and better memory (44).

Regarding motor-cognitive performance, we demonstrated that strobe training improves all motor-cognitive skills (cognitive agility, visual processing speed, and visual memory), with large effect sizes. Good (45) suggests that cognitive agility is the individual's capacity to mindfully oscillate between openness and focus, as a real-time adaptation to dynamic contexts. Cognitive agility helps perform well in dynamic decision-making contexts (45). Climbing is formed by dynamic moves where the action of going to the next hold needs making a decision about the route path. Vision and visual processing are important factors for successful athletic performance (34). The most common training methods for optimizing visual performance are refractive compensation, filters, nutrition, and sports vision training (34). An example of sport vision training is strobe training. Moreover, higher-level athletes detect better perceptual cues, make more efficient movements, and have a higher processing speed and higher level of attention in comparison to lower-level athletes (46). This argues that strobe training is a type of visual training meant for higher-level athletes. Sport vision programs explain that practicing with demanding visual, perceptual, and sensorimotor tasks will improve vision, which will lead to better sensory processing, better motor movements, and improved athletic performance (34). In many sports, vision is a key factor for successful performance.

Regarding climbing-specific parameters, we demonstrated that strobe training improves some climbing-specific variables: mainly for flexibility (foot raise with rotation and without rotation) and upper body strength (finger hang, push-up shoulder endurance, and dips), but with small and medium effect sizes. We believe this result is due to the additional climbing session that the athlete had to do weekly, because strobe training involves climbing exercises, just by increasing the training volume. The importance of general physical training (improving strength, aerobic capacity, coordination, and balance) and specific physical training (improving overall endurance, especially upper body strength on specific holds, flexibility, and ascending speed) for enhancing climbing performance was already proven by several studies (47–52).

Another important result came from the effect sizes of the types of performances enhanced by strobe training. Cognitive performance and motor-cognitive performance improved with a large effect size, while climbing-specific parameters improved with small and medium effect sizes. Moreover, climbing performance improved with a small effect size. This result explained that strobe training acts more on the cognitive component of training than on the motor component of training in climbing. A higher level of sports performance requires cognitive functions such as attention, decision making, and working memory (53). Cognitive training in sports is a highly researched method for enhancing cognitive performance, but it is still not known how it can transfer to athletic performance and more studies are needed to guide coaches and athletes to maximize training for performance (53).

One practical application of the research was extending the use of the Cognitrom Assessment technology and Witty SEM technology for evaluating performance in climbing. To the authors’ best knowledge, this is one of the first studies that used Cognitrom Assessment technology in evaluating climbers. We evaluated selected cognitive skills (spatial skills and reactivity) and analyzed their relation to climbing performance. Cognitrom Assessment technology is a wide test battery composed of cognitive tests, validated on the Romanian population, but has never been used to evaluate climbers.^5^ To the authors’ best knowledge, this is one of the first studies that used Witty SEM technology in evaluating climbers. We highlighted an evaluating protocol for climbers describing the setup of the semaphores and the evaluating tests. Witty SEM technology is a relatively new system that was previously used as an evaluation method (54–59) as well as a training method (60).

The second practical application of the research was extending the use of strobe technology for enhancing performance in climbing. To the authors’ best knowledge, this is one of the first studies that implemented strobe training in climbing on elite youth athletes and demonstrated that it improved performance by improving cognitive performance. Strobe training is a relatively new training method for all sports disciplines, being used only in a few, such as badminton (19), baseball (4, 20), cricket (21), football (22), ice hockey (17, 23), tennis (24, 25), and softball (27).

Our study has several strengths. The main strength comes from extending the use of the Cognitrom Assessment system and Witty SEM technology as evaluating methods for climbing. We also extended the use of strobe glasses as a visual training method for enhancing climbing performance. Another strength of the study was the research group, evaluating the best youth climbers spread all around Romania. In addition, because of the strict inclusion criteria, we considered our group to be homogenous, having similar experience, similar technical training, similar invested time in climbing, and similar nutrition habits. Moreover, we evaluated selected cognitive and motor-cognitive skills to be a climber's need, abilities that are often omitted in athlete training in comparison with physical, technical, or psychological abilities (61).

The present study has some limitations. First, the cognitive variables were measured in general; several assessment tools should be developed to evaluate climbing-specific cognition. Second, the climbing performance was recorded from their subjective history and on different routes (as their personal best), even though self-reporting grades are appropriate as climbers accurately self-report their climbing ability in research contexts (32). Future research should analyze the influence of strobe training on competitive performance, where all the climbers from the research group are in rivalry with the others and compete on the same route/routes. Another limitation came from the fact that we analyzed only climbers specialized in lead climbing and bouldering. We did not make any difference between male and female climbers. Another limitation came from the relatively wide age limit, analyzing climbers aged 13–20 years, a period of time when there can be wide individual differences in terms of cognitive and physical attributes (62). A further limitation of the study also came from not analyzing factors from the invisible training (nutrition habits, rest periods, recreational activities). Lastly, another limitation came from using the strobe technology, being a relatively new technology. To date, there is no article that tested the effects of strobe training depending on the level of expertise. The majority of articles focused on ball-tracking sports. Another impediment came from the worldwide limitation of sports research in terms of scale and subject participation. The published papers that evaluated strobe training had small sample sizes (less than 15 athletes), so the statistical analyses are difficult to implement, but the intention of a strobe intervention is to improve the performance of elite athletes (16).

## Data Availability

The raw data supporting the conclusions of this article will be made available by the authors, without undue reservation.

## Footnotes

^1^ https://www.cognitrom.ro/produs/evaluare-psihologica/ (accessed March 5.2024, 13:49).

^2^https://training.microgate.it/sites/default/files/manuali/Witty-UserManual-EN.pdf (accessed: January 4, 2024, 18:48).

^3^https://www.ircra.rocks/mct-documents (accessed February 20, 2024, 16:14).

^4^https://www.ualberta.ca/sport-system/media-library/ltad/climbing-ltad.pdf (accessed January 2, 2024, 11:27).

^5^https://www.cognitrom.ro/produs/evaluare-psihologica/ (accessed January 2, 2024, 15:07).
